# Self-Organized Criticality Theory of Autoimmunity

**DOI:** 10.1371/journal.pone.0008382

**Published:** 2009-12-31

**Authors:** Ken Tsumiyama, Yumi Miyazaki, Shunichi Shiozawa

**Affiliations:** 1 Department of Biophysics, Kobe University Graduate School of Health Science, Kobe, Japan; 2 Department of Medicine, Kobe University Graduate School of Medicine, Kobe, Japan; 3 The Center for Rheumatic Diseases, Kobe University Hospital, Kobe, Japan; 4 Global Center of Excellence (GCOE), Tokyo, Japan; New York University, United States of America

## Abstract

**Background:**

The cause of autoimmunity, which is unknown, is investigated from a different angle, i.e., the defect in immune ‘system’, to explain the cause of autoimmunity.

**Methodology/Principal Findings:**

Repeated immunization with antigen causes systemic autoimmunity in mice otherwise not prone to spontaneous autoimmune diseases. Overstimulation of CD4^+^ T cells led to the development of autoantibody-inducing CD4^+^ T (*ai*CD4^+^ T) cell which had undergone T cell receptor (TCR) revision and was capable of inducing autoantibodies. The *ai*CD4^+^ T cell was induced by *de novo* TCR revision but not by cross-reaction, and subsequently overstimulated CD8^+^ T cells, driving them to become antigen-specific cytotoxic T lymphocytes (CTL). These CTLs could be further matured by antigen cross-presentation, after which they caused autoimmune tissue injury akin to systemic lupus erythematosus (SLE).

**Conclusions/Significance:**

Systemic autoimmunity appears to be the inevitable consequence of over-stimulating the host's immune ‘system’ by repeated immunization with antigen, to the levels that surpass system's self-organized criticality.

## Introduction

Since ‘clonal selection theory of immunity’ of F. Macfarlane Burnet and subsequent molecular biological discoveries on V(D)J recombination and the diversity and individuality of immune response, how autoimmunity arises remains unclear. Apart from the term ‘autoimmunity’ which is now ready-made, in the present study, we tried to see the pathogenesis of autoimmunity from different angle and test the integrity of immune ‘system’. The method we have chosen was to stimulate the system maximally by antigen to the levels far beyond its steady-state just like testing the capability of automobile. In a perfectly reproducible experiments in which the mice not prone to autoimmune diseases were immunized repeatedly with antigen, we have unexpectedly and surprisingly discovered that overstimulation of immune system beyond its self-organized criticality inevitably leads to systemic autoimmunity. Subsequent detailed molecular analyses revealed in the first that autoantibodies are induced not by cross reaction to antigen but by *de novo* T cell receptor (TCR) revision. Second, final maturation of effector cytotoxic T lymphocyte (CTL) *via* antigen cross-presentation is *sine qua non* for generating autoimmune tissue injury. Most importantly, we now show that autoimmunity arises not from ‘autoimmunity’, but as a natural consequence of normal immune response when stimulated maximally beyond system's self-organized criticality.

## Results

### Induction of Autoantibodies

**Figure 1:**
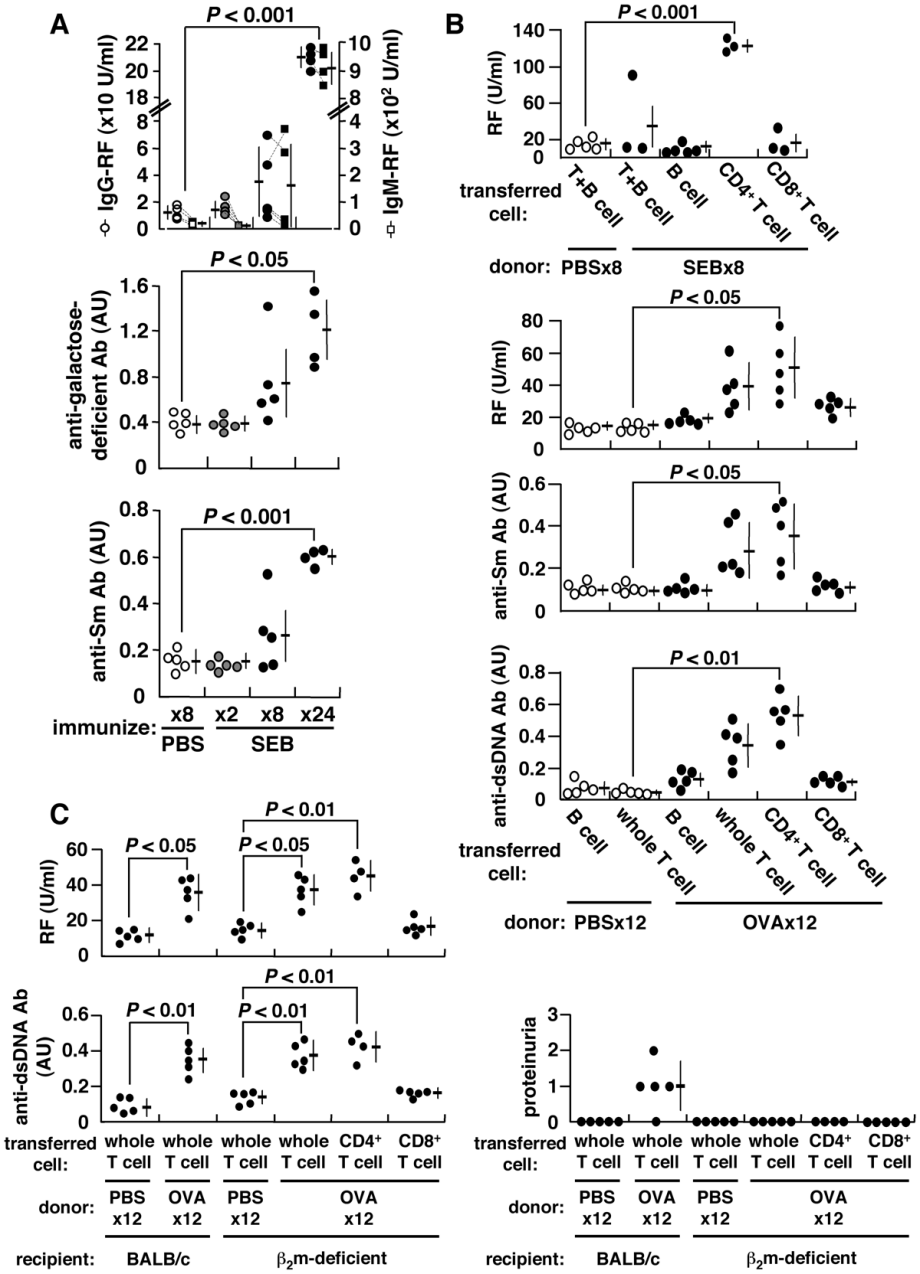
Induction of autoantibodies and proteinuria. BALB/c mice were repeatedly injected i.p. with 25 µg SEB, 500 µg OVA or PBS every 5 d. (A) Serum IgG- and IgM-RFs, anti-galactose-deficient IgG and anti-Sm antibodies were measured using ELISA. The arbitrary unit (AU) of 1.0 is equivalent to the titer obtained from sera of prototypic autoimmune MRL/lpr mice. Data from each mouse are connected by dotted lines. (B) Adoptive transfer of splenic B, T, CD4^+^ T or CD8^+^ T cells of SEB-, OVA- or PBS-immunized BALB/c mice into naïve BALB/c mice. The recipient mice were given single i.p. injection of 25 µg SEB or 500 µg OVA 24 h after cell transfer, and autoantibodies were measured 2 weeks later. (C) Adoptive transfer of cells from OVA-immunized BALB/c mice into β_2_m-deficient mice.

Consistent with the common observation that T cells become anergic after strong stimulation with antigen [1], we observed that 2× immunization with staphylococcus enterotoxin B (SEB) caused SEB-reactive Vβ8^+^CD4^+^ T cells from BALB/c mice to become anergized. However, these cells recovered from anergy to divide and produce IL-2 after further immunization 8× with SEB (Figure S1A). This was accompanied by the induction of autoantibodies, including IgG- and IgM-rheumatoid factor (RF), anti-Sm antibody, and in particular, RF reactive against galactose-deficient IgG, typically found in human autoimmunity [2] (Figure 1A). Autoantibodies can also be induced by other conventional antigens, including ovalbumin (OVA) or keyhole limpet hemocyanin (KLH) () as long as immunizing antigen is correctly presented to T cells (Figure S1B). CD4^+^ T cells of repeatedly-immunized mice become fully matured, expressing CD45RB^lo^, CD27^lo^ and CD122^hi^ (data not shown), and these primed CD4^+^ T cells can confer RF generation in naïve recipients following adoptive transfer (Figure 1B). The induction of autoantibodies is independent of CD8^+^ T cells or MHC class I-restricted antigen presentation for the following reasons. First, both RF and anti-dsDNA antibody can be consistently induced upon repeated immunization of β_2_-microglobulin (β_2_m)-deficient BALB/c mice with OVA. β_2_m-deficient mice are deficient in CD8^+^ T cells, which are reduced to <0.8% of splenic T cells [3] (Figure S3). Second, the ability to induce autoantibodies was transferable from OVA-immunized BALB/c mice to β_2_m-deficient mice solely *via* CD4^+^ T cells (Figure 1C). Thus, CD4^+^ T cells from repeatedly-immunized mice acquire the ability to induce autoantibodies. We refer to these as autoantibody-inducing CD4^+^ T (*ai*CD4^+^ T) cells in this communication.

### Mechanism of Autoantibody Induction

**Figure 2:**
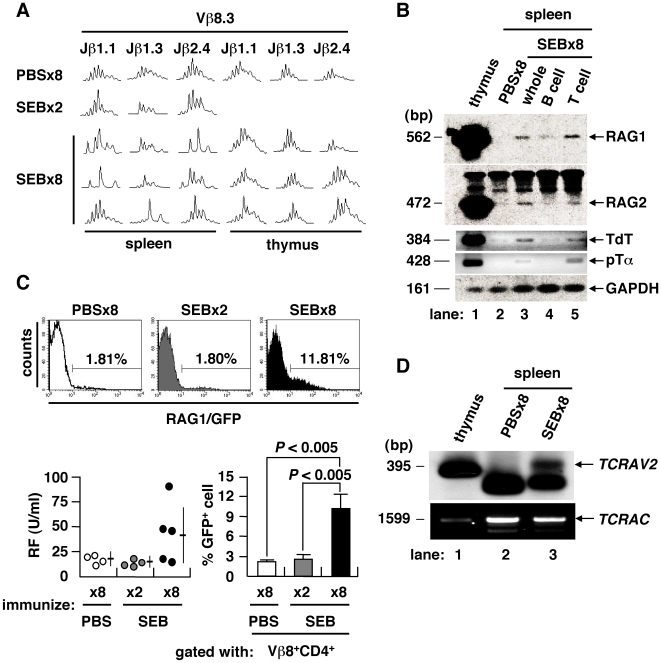
TCR revision upon repeated immunization with antigen. (A) TCR CDR3 length profiles of mice immunized 8× with PBS, 2× or 8× with SEB. TCR repertoire of splenic CD4^+^ T cell was skewed only after immunization 8× with SEB. (B) Expression of V(D)J recombinase complex and related molecules in the spleen of PBS- or SEB-injected BALB/c mice. (C) GFP^+^ cells in the Vβ8^+^CD4^+^ T population of *rag1/gfp* knock-in mice. IgG-RF as induced in *rag1/gfp* knock-in mice after immunization 8× with SEB (lower left). The GFP^+^ T cell fraction was also increased among Vβ8^+^CD4^+^ T cells (mean ± SD, 4–5 mice/group). (D) TCRα chain revision in the spleen of mice immunized 8× with SEB was determined by LM-PCR detection of dsDNA breaks at the RSS flanking the *TCRAV2*, with PCR-amplified TCRα constant region (*TCRAC*) as a DNA quality control.

To further clarify the characteristics of *ai*CD4^+^ T cells, we examined their TCR repertoire by spectratyping of their complementarity determining region 3 (CDR3) [4]. Combinatorial assessment of Vβ and Jβ showed that the CDR3 length profiles of CD4^+^ splenocytes in mice immunized either 8× with PBS or 2× with SEB fit a normal Gaussian curve, typical of a diverse and unbiased TCR repertoire (Figure 2A). However, splenocytes, but not thymocytes, from mice immunized 8× with SEB showed skewed length profiles, suggesting that TCR revision was in progress at periphery of the spleen. Genes encoding components of the V(D)J recombinase complex were specifically re-expressed in mice immunized 8× with SEB, including the recombination-activating genes 1 and 2 (RAG1/2), terminal deoxynucleotidyl transferase (TdT) and surrogate TCRα chain (pTα) [5] (Figure 2B). The RAG1 gene is expressed *in vivo* after immunization 8× with SEB in *rag1/gfp* knock-in mice [6] (Figure 2C). In these mice, serum RF was increased in conjunction with an increase of GFP-expressing Vβ8^+^CD4^+^ T cells in the spleen. To directly prove that V(D)J recombination took place at the periphery in spleen, we used ligation-mediated PCR (LM-PCR) to detect blunt-end DNA fragments harboring a rearranged coding V region flanked by recombination signal sequences (RSS) [7], [8]. We identified rearranged intermediates corresponding to the TCRα variable region 2 (*TCRAV2*) in the splenocytes of mice immunized 8× with SEB (Figure 2D). These findings indicate that repeated immunization with conventional antigen can induce the generation of *ai*CD4^+^ T cells which have undergone TCR revision and are capable of stimulating B cells [9]. This observation is in line with previous findings showing that such somatic mutations occur often in lymphocytes, a process which is considered to be a major stochastic element in the pathogenesis of autoimmunity [10], [11]. Thus, overstimulation of CD4^+^ T cells by repeated immunization with antigen and induction of full maturation inevitably leads to the generation of *ai*CD4^+^ T cells which have undergone TCR revision and are capable of inducing autoantibodies. Importantly, the present study shows that such *ai*CD4^+^ T cells are induced by *de novo* TCR revision but not by cross-reaction to antigen.

### Induction of Autoimmune Tissue Injury

**Figure 3:**
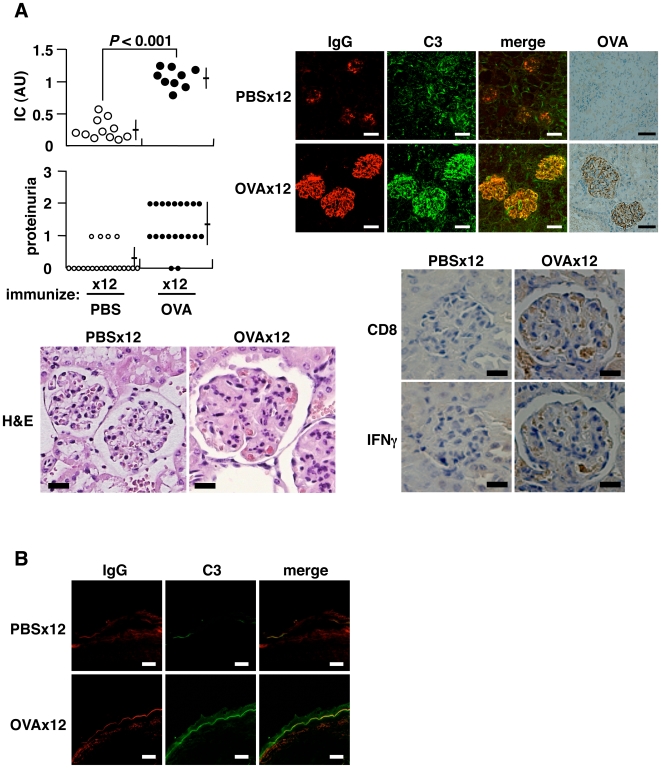
Induction of autoimmune tissue injury. BALB/c mice were injected i.p. with 500 µg OVA every 5 d. (A) Serum IC measured 2 d after final immunization, expressed as AU. Proteinuria assessed 9 d after final immunization: grades 1, 2 and 3 represent 30–100 mg/dl, 100–300 mg/dl and 300–1000 mg/dl of urinary protein, respectively (upper left). Representative histopathology of kidneys from mice immunized 12× with PBS or OVA (lower left) (H&E staining, bar  = 20 µm; original magnification ×400): glomerular expansion with cellular infiltration including eosinophils seen under the same magnification. Immunohistochemistry for deposited IC, IgG, C3 and OVA (upper right) (bar  = 50 µm; original magnification ×200), and cells infiltrated into glomeruli (bar  = 20 µm; original magnification ×300), stained in serial tissue sections using anti-CD8α (53–6.7) and anti-IFNγ (R4-6A2) monoclonal antibodies, in the specimens of mice immunized 12× with OVA (lower right). (B) Lupus band test stained with anti-IgG and anti-C3 antibodies (bar  = 20 µm; original magnification ×400).

Repeated immunization with OVA can also lead to autoimmune tissue injury and the production of autoantibodies reactive against IgG, Sm and dsDNA (Figure 3 and Figure S2A). Serum immune complex (IC), proteinuria, and the deposition of IC and OVA in the kidney were noted in mice immunized 12× with OVA (Figure 3A). Typical diffuse proliferative glomerular lesions were seen in the kidney, and these glomeruli were infiltrated with CD8^+^ T cells. These observations resemble the clinical features observed in lupus patients, who typically exhibit an increase in CD8^+^ T cells in the peripheral blood and infiltration of CD8^+^ T cells in kidney [12], [13]. Immunization of mice 12× with OVA led to re-expression of the V(D)J recombinase complex and enlargement of the spleen (Figure S4A), and an increase in anti-dsDNA antibody, which is uniquely linked to autoimmune tissue injury in lupus nephritis [14] (Figure S2A). Pathological findings included diffuse membranous (wire-loop) and/or proliferative glomerulonephritis in the kidney (Figure 3A), infiltration of plasma cells around hepatic bile ducts (Figure S4B), enlarged lymphoid follicles with marked germinal center in spleen (Figure S4B), occasional lymphocyte infiltration into the salivary glands (data not shown), lymphoid cell infiltration into the thyroid, and perivascular infiltration of neutrophils and macrophages into the skin dermis of the auricle (Figure S4B). The lupus band test, diagnostic of SLE, was positive in the skin at the epidermal-dermal junction (Figure 3B).

### Mechanism of Autoimmune Tissue Injury

**Figure 4:**
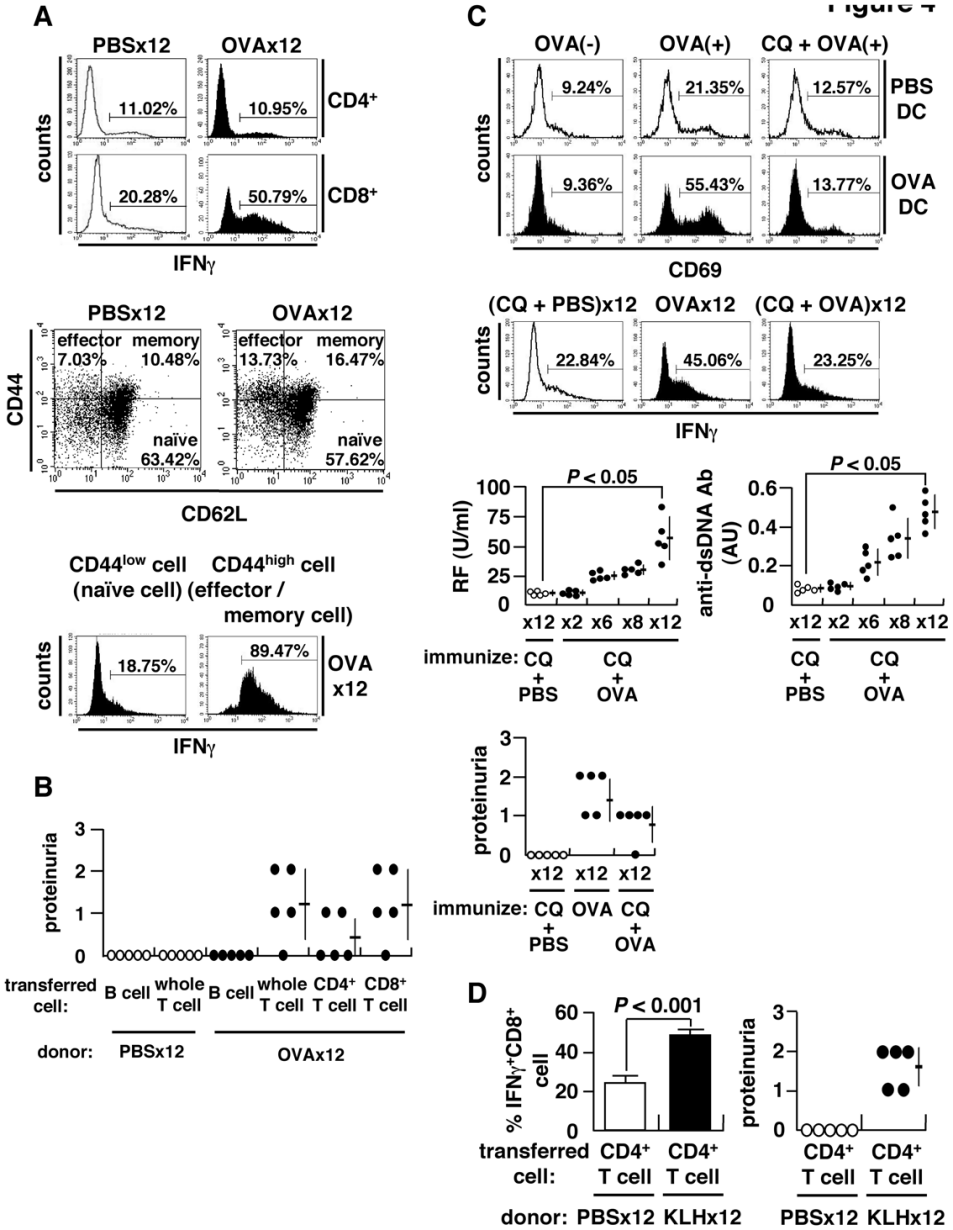
Expansion of CD8^+^ T cells and antigen cross-presentation. (A) Spleen cells stimulated with 50 ng/ml phorbol myristate acetate (PMA) and 500 ng/ml ionomycin for 4 h in the presence of brefeldin A (10 µg/ml) and stained for intracellular IFNγ (upper). Subsets of CD8^+^ T cells categorized into naïve (CD44^low^CD62L^high^), effector (CD44^high^CD62L^low^), and memory (CD44^high^CD62L^high^) fractions (middle). Flow cytometry of IFNγ^+^ cells within naïve or effector/memory CD8^+^ T cell populations. Spleen cells were separated into naïve (CD44^low^) and effector/memory (CD44^high^) cells using CD44 MACS beads, and IFNγ^+^ cells within the CD8^+^ T population was evaluated (lower). (B) Adoptive transfer of splenocytes of OVA-immunized BALB/c mice into naïve recipients. The recipients were injected with 500 µg OVA 24 h after cell transfer, and proteinuria examined 2 weeks later. (C) Cross-presentation of OVA to CD8^+^ T cells. Splenic CD11c^+^ DC from OVA-immunized or control mice were incubated in the presence (OVA(+)) or absence (OVA(−)) of 1 mg/ml OVA with or without chloroquine (CQ) (20 µg/ml) for 3 h, followed by a co-culture with KJ1-26^+^CD8^+^ T cells of DO11.10 transgenic mice for 24 h to examine surface expression of CD69 (upper). Inhibition of cross-presentation *in vivo* by administration of 250 µg CQ per mouse 3 h prior to immunization with OVA or PBS. IFNγ^+^CD8^+^ T cells (middle), autoantibodies and proteinuria (lower) after 12× immunization. (D) Requirement of autoantibody-inducing CD4^+^ T cells for CD8^+^ T cell-mediated autoimmune tissue injury. BALB/c mice were immunized 12× with KLH, and CD4^+^ T cells were isolated using MACS beads. Cells were transferred into the anti-CD4 antibody-treated recipient mice immunized 8× with OVA. Percent matured CTL, i.e., IFNγ^+^CD8^+^ T cells, and proteinuria were measured 2 weeks after booster immunization 1× with KLH.

It has been shown previously that IFNγ is increased in association with autoimmune tissue injury [15]–[17]. Consistent with this, we found that the number of IFNγ^+^CD8^+^ T cells, but not regulatory T or T helper 17 cells, was increased following immunization 12× with OVA (Figure 4A and data not shown). We also observed an expansion of IFNγ-producing effector/memory CD8^+^ T cells, which are necessary for adaptive immunity [18] (Figure 4A). These IFNγ-producing CD8^+^ T cells were observed to have infiltrated into OVA-deposited glomeruli of OVA-immunized mice (Figure 3A). CD8^+^ T cells are required for tissue injury based on the following observations. First, the transfer of CD8^+^ T cells can induce renal lesions in mice (Figure 4B), as well as the generation of new IFNγ^+^CD8^+^ T cells in the spleens of recipient mice following cell transfer (Figure S5). Second, autoimmune tissue injury is not induced by the transfer of CD8^+^ T cells from OVA-immunized wild-type mice into β_2_m-deficient mice (Figure 1C). And finally, CD8^+^ T cell transfer must be accompanied by at least a 1× booster immunization with OVA to induce autoimmune tissue injury in the recipient mice (Figure S6). The findings indicate that full-matured, IFNγ-producing effector CD8^+^ T cells are required for the induction of autoimmune tissue injury, provided that the relevant antigen is correctly presented on the target organs. These are well-established characteristic of CTL and not novel. We show, however, that (i) CTL is induced through an immune, but not ‘autoimmune’, process, and that (ii) autoimmune tissue injury inevitably occurs when CD8^+^ T cells are overstimulated to become matured effector CTLs. The latter means that regardless of how CTL is induced, the consequence of CTL over-induction is immune tissue injury.

### Antigen Cross-Presentation

We next show that antigen cross-presentation is required for the induction of CTL and tissue injury. To test this, we co-cultured OVA-pulsed dendritic cells (DC) from mice immunized 12× with OVA together with T cells from OVA-TCR transgenic DO11.10 mice exclusively expressing OVA-reactive TCR [19]. We show that OVA-reactive DO11.10 CD8^+^ T cells are activated upon co-culture with OVA-pulsed DCs (Figure 4C and Figure S7). Further, autoimmune tissue injury and the increase in IFNγ^+^CD8^+^ T cells, but not of autoantibody generation, were both abrogated by adding chloroquine (CQ), an inhibitor of antigen cross-presentation (Figure 4C). This indicates that antigen cross-presentation is required for the expansion of IFNγ-producing CD8^+^ T cells and autoimmune tissue injury.

### *ai*CD4^+^ T Cell Helps CD8^+^ T Cell to Induce Tissue Injury

Since CTL appear to play a rather passive role in autoimmunity, we next studied whether or not *ai*CD4^+^ T cell help is required for the induction of autoimmune tissue injury. Since anti-CD4 treatment almost abrogates generation of IFNγ-producing CD8^+^ T cell and autoimmune tissue injury in OVA-immunized BALB/c mice (Figure S8), to test whether this CD4^+^ T cell-mediated help is mediated by *ai*T cells or antigen-specific T cells, we have transferred CD4^+^ T cells from mice immunized 12× with KLH into CD4^+^ T-depleted BALB/c mice immunized 8× with OVA (Figure 4D). Because full-matured IFNγ^+^ CTLs do not develop with less than 8× immunization with OVA (Figure S9), this experiment can test the ability of *ai*CD4^+^ T cells that have undergone TCR revision to promote the maturation of OVA-specific CTL. The result showed that both autoimmune tissue injury and OVA-specific IFNγ^+^CD8^+^ T cells arose in these mice after transfer, indicating that *ai*CD4^+^ T cells with *de novo* TCR revision are required for the maturation of CD8^+^ T cell and autoimmune tissue injury (Figure 4D).

## Discussion

The present findings are consistent with the current consensus that CD4^+^ T cells normally die *via* activation-induced cell death (AICD) after repeated exposure to a single antigen, while naïve CD4^+^ T cells having a ‘cross-reactive’ TCR with lower affinity can be activated through repeated exposure to the same antigen and survive due to weak TCR signaling, ultimately acquiring autoreactivity [20]. We show here, however, that *ai*CD4^+^ T cells are induced not by cross-reaction, but by *de novo* TCR revision. The *ai*CD4^+^ T cells thus generated induce not only autoantibodies but also full-maturation of CD8^+^ T cells leading to autoimmune tissue injury akin to human SLE. Thus, induction of *ai*CD4^+^ T cells is a critical step, and subsequent induction of effector CTL is a critical next step in the development of autoimmunity [21], [22]. The question of how autoimmunity is triggered can therefore be deduced to the quantitative response of host against immunizing antigen, i.e., the ability of host to present and/or cross-present antigen. It then follows that the ability of certain antigens such as measles virus to cause autoimmunity may be due to their ability, in conjunction with its ability to present antigen, to overstimulate CD4^+^ and/or CD8^+^ T cells of certain hosts beyond integrity of their immune system. Living organisms are constantly exposed to a broad range of environmental antigens, as exemplified by the recent re-emergence of measles virus infection among a subpopulation of Japanese young adults who were not vaccinated against the virus. We therefore conclude that systemic autoimmunity necessarily takes place when host's immune ‘system’ is overstimulated by external disturbance, i.e., repeated exposure to antigen, to the levels that surpass system's self-organized criticality, and propose here ‘self-organized criticality theory’ explaining the cause of autoimmunity.

## Materials and Methods

### Ethics Statement

This study was approved by the Institutional Animal Care and Use Committee and carried out according to the Kobe University Animal Experimental Regulations.

### Reagents

APC (allophycocyanin)-conjugated antibody against CD4 (RM4-5), and PE-conjugated antibodies against CD62L (MEL-14), CD69 (H1.2F3) and were purchased from BioLegend (San Diego, CA); FITC-conjugated antibodies against CD44 (IM7.8.1) and DO 11.10 clonotypic TCR (KJ1-26) and PE-conjugated rat IgG1 isotype control from CALTAG Laboratories (Burlingame, CA); PE-Cy5 (phycoerythrin-cyanin 5)-conjugated antibody against CD8α (53-6.7), PE-conjugated antibodies against Vβ8 TCR (F23.1) and IFNγ (XMG1.2) from BD PharMingen (San Diego, CA).

### Animal Studies

Animal studies with BALB/c female mice (Japan SLC, Inc., Hamamatsu, Japan) and DO11.10 TCR transgenic mice [19] (Jackson Laboratory, Bar Harbor, ME), β_2_m-deficient mice [3] and *rag1/gfp* knock-in mice [6] of BALB/c background were performed with the approval of the Institutional Review Board. Mice (8 weeks-old) were immunized with 25 µg SEB (Toxin Technologies, Sarasota, FL), 500 µg OVA (grade V; Sigma, St. Louis, MO), 100 µg KLH (Sigma) or PBS by means of i.p. injection every 5 d.

Frozen sections of kidney and dermis were stained for C3, IgG or OVA using goat anti-C3 (Bethyl laboratories, Inc., Montgomery, TX) and Alexa Fluor 488-conjugated anti-goat IgG antibodies (Molecular Probes, Eugene, OR), Alexa Fluor 594-conjugated anti-mouse IgG antibody (Molecular Probes), or rabbit anti-OVA antibody (Sigma). For CD8 or IFNγ staining, paraffin-embedded sections of kidney were stained with rat antibodies against CD8α (53-6.7; BD PharMingen) or IFNγ (R4-6A2; BD PharMingen), followed by reaction with VECTASTAIN Elite ABC rat IgG kit (Vector, Burlingame, CA).

To detect intracellular IFNγ, cells (1×10^6^/ml) were stimulated with 50 ng/ml phorbol myristate acetate (PMA; Sigma) and 500 ng/ml ionomycin (Sigma) in the presence of brefeldin A (10 µg/ml; Sigma). After 4 h, cells were stained with anti-CD8 antibody, followed by fixation with 2% formaldehyde, permeabilization with 0.5% saponin (Sigma) and stained for IFNγ.

For adoptive cell transfer, B, T, CD4^+^ T and CD8^+^ T cells were isolated from spleens to >90% purity using MACS beads (Miltenyi Biotec, Germany). The cells were transferred into naïve BALB/c or β_2_m-deficient mice *via* i.p. (5×10^6^/mouse) or i.v. (2.5×10^7^/mouse) injection. The recipients received a single i.p. injection of 25 µg SEB or 500 µg OVA 24 h after cell transfer, and sera, urine and organ of recipients were studied 2 weeks afterwards.

BALB/c mice were injected i.p. with 200 µg anti-CD4 antibody (GK1.5; BioLegend) to deplete CD4^+^ T cell 24 h after immunization 8× with OVA. Four days later, CD4^+^ T cells from mice immunized 12× with KLH were transferred to the CD4^+^ T-depleted mice. The recipient mice received a single i.p. injection of 100 µg KLH 24 h after the cell transfer.

### Assay for Mediators

Sera were assayed for anti-Sm antibody using Sm antigen (ImmunoVision, Springdale, AR), RF (Shibayagi Co., Gunma, Japan), RF for galactose-deficient IgG (Eisai Co., Ltd., Tokyo, Japan) and anti-dsDNA antibody using dsDNA (Worthington Biochemical Co., Lakewood, NJ) after digestion by S1 nuclease (Promega, Madison, WI). Serum IC was detected using goat anti-C3 antibody (Bethyl Lab.).

### CDR3 Length Spectratyping

cDNAs from thymocytes and CD4^+^ splenocytes were subjected to PCR amplification using Cβ- and Vβ8-specific primers. Amplified products were subjected to run-off reactions using three fluorophore-labeled Jβ primers, Jβ1.1, Jβ1.3 and Jβ2.4, and analyzed by GeneScan software (Perkin-Elmer Applied Biosystems, Emeryville, CA) [4].

### RT-PCR

Total RNA was reversely transcribed to cDNA and amplified by PCR [23]. The products were fractionated by electrophoresis and transferred to nylon membranes (Roche Diagnostics, Mannheim, Germany). The membranes were hybridized to fluorescein end-labeled probes and visualized by alkaline phosphatase (ALP)-labeled anti-fluorescein antibody and Gene Images CDP-Star chemiluminescence reaction (Amersham Pharmacia Biotech, Piscataway, NJ). The primers and probes were: 5′-CCAAGCTGCAGACATTCTAGCACTC-3′ (forward), 5′-CAACATCTGCCTTCACGTCGATCC-3′ (reverse) and 5′-AACATGGCTGCCTCCTTGCCGTCTACCCT-3′ (probe) for RAG1 [24]; 5′-CACATCCACAAGCAGGAAGTACAC-3′ (forward), 5′-GGTTCAGGGACATCTCCTACTAAG-3′ (reverse) and 5′-GCAATCTTCTCTAAAGATTCCTGCTACCT-3′ (probe) for RAG2 [24]; 5′-GAACAACTCGAAGAGCCTTCC-3′ (forward), 5′-CAAGGGCATCCGTGAATAGTTG-3′ (reverse) and 5′-ATTCGGTCACCCACATTGTGGCAGAGAAC-3′ (probe) for TdT; 5′-CAACTGGGTCATGCTTCTCC-3′ (forward), 5′-TGGCTGTCGAAGATTCCC-3′ (reverse) and 5′-CCGTCTCTGGCTCCACCCATCACACTGCT-3′ (probe) for pTα.

### LM-PCR

DNA (1 µg) was ligated to 20 µM BW linker using T4 ligase (Takara Bio Inc., Shiga, Japan) [25]. Primary PCR was performed using 200 ng ligated DNA, BW-1HR primer (5′-CCGGGAGATCTGAATTCGTGT-3′) [24], primer specific for 3′ flanking sequence of *TCRAV2* (5′-AGATGATACAGAGACAAAATGTGAGC-3′) and 2 U of AmpliTaq Gold DNA polymerase (Applied Biosystems, Foster City, CA). A second PCR was performed using 1 µl of the first PCR product (diluted 1/100), BW-1HR, and nested primer specific for 3′ flanking sequence of *TCRAV2* (5′-TATTGTGGATGCTAACAAGTGCTTTC-3′). Amplified DNA was transferred to membranes and visualized using fluorescein end-labeled probe specific for *TCRAV2* (5′-TAACATAAGAATGCACCGCTTACACC-3′) and ALP-labeled anti-fluorescein antibody. Primers for control *TCRAC* region were amplified using the primers 5′-CAGAACCCAGAACCTGCTGTG-3′ and 5′-ACGTGGCATCACAGGGAA-3′. Nomenclature of the *TCRA* gene segments was according to the ImMunoGeneTics (IMGT) database (http://imgt.cines.fr).

### Antigen Cross-Presentation

OVA-reactive CD8^+^ T cells were isolated from spleens of DO 11.10 mice using MACS beads (Miltenyi Biotec). CD11c^+^ DCs (4×10^5^/well) were isolated using MACS beads (Miltenyi Biotec) and incubated with 1 mg/ml OVA for 3 h, then co-cultured with DO11.10 CD8^+^ T (KJ1-26^+^CD8^+^) cells (2×10^5^/well) for 24 h, and the expression of CD69 on DO11.10 CD8^+^ T cells was examined. IL-2 and IFNγ in culture supernatants were measured by ELISA (Biosource, Camarillo, CA).

To inhibit cross-presentation, mice were immunized *in vivo* with 250 µg of chloroquine (Sigma) 3 h prior to immunization with 500 µg OVA or PBS every 5 d. Presence of autoantibodies was analyzed 2 d after each immunization, and proteinuria and IFNγ^+^CD8^+^ T cells were examined 9 d after the final immunization.

### Statistical Analysis

Statistical analyses were performed using Student's *t* test, and the data are expressed as the mean ± SD.

## Supporting Information

Figure S1 Induction of autoantibodies depends on correct presentation of antigen to T cells. (A) BALB/c mice were repeatedly injected i.p. with 25 µg of SEB or PBS every 5 d. Sorted Vβ8^+^CD4^+^splenocytes obtained 9 d after the final immunization were stimulated *in vitro* with plate-bound 2 µg/ml anti-CD3 (145-2C11; Cederlane, Ontario, Canada) and 5 µg/ml anti-CD28 (37.51; BD PharMingen) antibodies for 24 h. Culture supernatant assayed for IL-2 (mean ± SD, 5 mice/group), or the cells were labeled with carboxyfluorescein diacetate succinimidyl ester (CFSE; Molecular Probes) and further cultured for 72 h followed by flow cytometry. (B) Requirement of correct antigen presentation for induction of RF. Induction of RF after immunization 8× with SEB in B10.D2 and BALB/c mice (efficient in presenting SEB) and in C57BL/6 (B6) mice (inefficient in presenting SEB).
(1.17 MB TIF)

Figure S2 Generation of autoantibodies after repeated immunization with antigen. (A) The 8 week-old BALB/c mice were injected i.p. with 500 µg OVA every 5 d, and serum RF and anti-Sm, and anti-dsDNA antibodies (upper), and serum IgG and anti-OVA antibodies (lower) were quantified by ELISA 2 d after respective immunization. An arbitrary unit (AU) of 1.0 is the equivalent titer in sera of MRL/lpr mice. Serum IgG was quantified by ELISA (Bethyl Laboratories), and anti-OVA antibody was quantified using mouse anti-OVA monoclonal antibody (OVA-14; Sigma) as reference. (B) BALB/c mice were immunized i.p. with 100 µg KLH every 5 d. Serum RF and anti-Sm antibodies were measured by ELISA 2 d after respective immunization, AU 1.0 =  equivalent detected in sera of MRL/lpr mice.
(1.00 MB TIF)

Figure S3 Induction of autoantibodies in CD8^+^ T cell-deficient mice. β_2_m-deficient mice were immunized with 500 µg OVA *via* i.p. injection every 5 d, and IgG-RF, anti-dsDNA antibody, and proteinuria were measured.
(0.69 MB TIF)

Figure S4 Expression of V(D)J recombinase complex and histopathology of OVA-immunized BALB/c mice. (A) Expression of V(D)J recombinase complex after immunization 12× with OVA as detected using RT-PCR (upper left). GFP^+^ cells in the CD4^+^ T cell of *rag1/gfp* knock-in mice after immunization 12× with OVA (lower left). Appearance and weights of spleens and a representative low-magnification view of the spleens from PBS- and OVA-immunized mice (right, mean ± SD, 9 mice/group). Enlarged lymphoid follicles with marked germinal centers were seen in mice immunized with OVA (H&E staining, bar  = 200 µm; original magnification ×20). (B) Representative renal and extra-renal histopathology in the mice immunized 12× with OVA. A wire-loop-like massive membranous glomerulonephritis in the kidney (upper left) (PAS staining, bar  = 20 µm; original magnification ×400), plasma cell infiltrates around bile ducts (upper middle) (bar  = 20 µm; original magnification ×400), expansion of lymphoid follicle in the white pulp of spleen (upper right) (bar  = 200 µm; original magnification ×40), focal infiltrates of mononuclear cells to thyroid (lower left) (bar  = 50 µm; original magnification ×100), and diffuse infiltration of inflammatory cells into auricular subcutaneous tissue (upper right) (bar  = 50 µm; original magnification ×200).
(6.01 MB TIF)

Figure S5 The *de novo* generation of IFNγ-producing CD8^+^ T cells in recipient mice after cell transfer. Percentage of IFNγ^+^ cells within the CD8^+^ T population of the recipient mice was examined 2 weeks after cell transfer (mean ± SD, 5 mice/group).
(0.73 MB TIF)

Figure S6 Transfer of the ability to induce anti-ds DNA antibody or tissue injury by transfer of CD4^+^ or CD8^+^ T cells, respectively. Adoptive transfer of cells from OVA-immunized mice into naïve BALB/c mice, with or without 1× booster injection of OVA (500 µg, 24 h post-transfer). Autoantibodies and proteinuria measured 2 weeks later.
(0.70 MB TIF)

Figure S7 Antigen-specific activation of T cells and the expression of MHC class I on DC. (A) Spleen cells were cultured with or without 1 mg/ml of OVA for 24 h, and the expression of CD69 on CD4^+^ T or CD8^+^ T cells was examined by flow cytometry. (B) DC from PBS- or OVA-immunized mice (PBS DC or OVA DC) were incubated in the presence or absence of chloroquine (CQ) (20 µg/ml) for 2 h and OVA (1 mg/ml) for 3h. OVA- and/or CQ-pulsed DCs were stained with biotin-conjugated anti-H-2k^d^ antibody (SF1-1.1; BD PharMingen) and PE-conjugated streptavidin (BioLegend).
(1.62 MB TIF)

Figure S8 Requirement of CD4^+^ T cell help for inducing autoimmune tissue injury. The mice were depleted of CD4^+^ T cells by treatment with 200 µg anti-CD4 antibody (Ab) (GK1.5; BioLegend) 24 h prior to 6×, 9× and 12× immunization with OVA. Control mice were injected with 200 µg rat IgG (CALTAG Lab.). (A) A representative flow cytometry plot showing that CD4+ T cells were depleted to 5.56±2.30% in the spleen and 3.42±1.02% in peripheral blood mononuclear cells (PBMC) 9 d after 3rd treatment with anti-CD4 Ab. (B) Mice were immunized 12× with OVA with or without adding anti-CD4 antibodies, and the number of IFNγ^+^ cells within the CD8^+^ T population (upper and lower left) (mean ± SD, 5 mice/group) and proteinuria (lower right) were evaluated.
(2.02 MB TIF)

Figure S9 Study on the requirement of autoantibody-inducing CD4^+^ T cells for autoimmune tissue injury. Neither OVA-specific matured IFNγ^+^CD8^+^ T cells or autoimmune tissue injury were observed until BALB/c mice were immunized at least 10× with OVA. The percent splenic IFNγ^+^CD8^+^ T cells (left, mean ± SD, 4 or 5 mice/group) and proteinuria (right) were examined after immunization 6×, 8×, 10× and 12× with OVA.
(0.67 MB TIF)
