# Supplementary figures and images for: Self-Organized Criticality Theory of Autoimmunity
Source: PLoS One. 2009 Dec 31;4(12):e8382. doi: 10.1371/journal.pone.0008382 (PMC2795160; doi:10.1371/journal.pone.0008382)

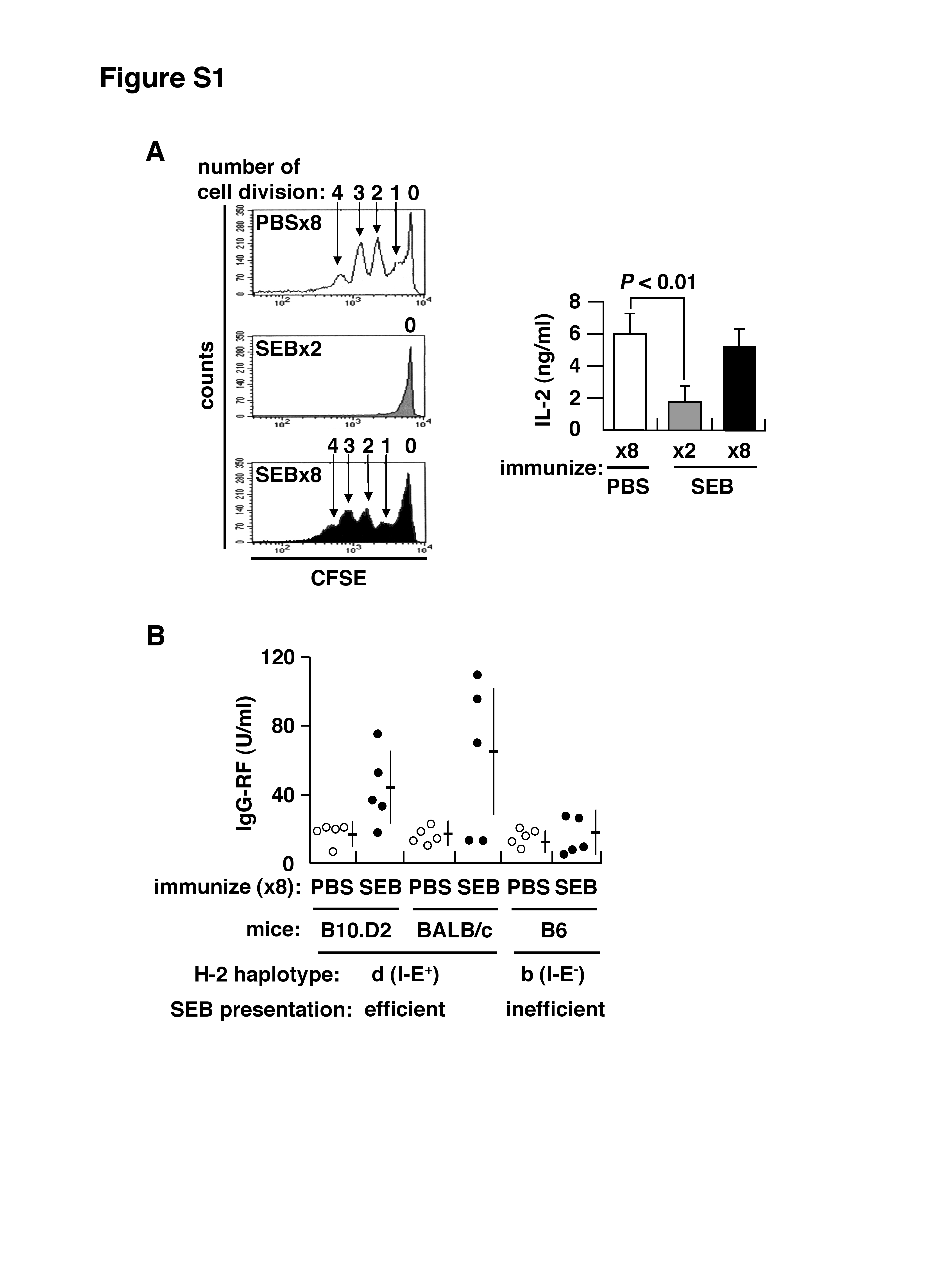

Supplement: Figure S1 — Induction of autoantibodies depends on correct presentation of antigen to T cells. (A) BALB/c mice were repeatedly injected i.p. with 25 µg of SEB or PBS every 5 d. Sorted Vβ8+CD4+splenocytes obtained 9 d after the final immunization were stimulated in vitro with plate-bound 2 µg/ml anti-CD3 (145-2C11; Cederlane, Ontario, Canada) and 5 µg/ml anti-CD28 (37.51; BD PharMingen) antibodies for 24 h. Culture supernatant assayed for IL-2 (mean ± SD, 5 mice/group), or the cells were labeled with carboxyfluorescein diacetate succinimidyl ester (CFSE; Molecular Probes) and further cultured for 72 h followed by flow cytometry. (B) Requirement of correct antigen presentation for induction of RF. Induction of RF after immunization 8× with SEB in B10.D2 and BALB/c mice (efficient in presenting SEB) and in C57BL/6 (B6) mice (inefficient in presenting SEB). (1.17 MB TIF) [file pone.0008382.s001.tif]

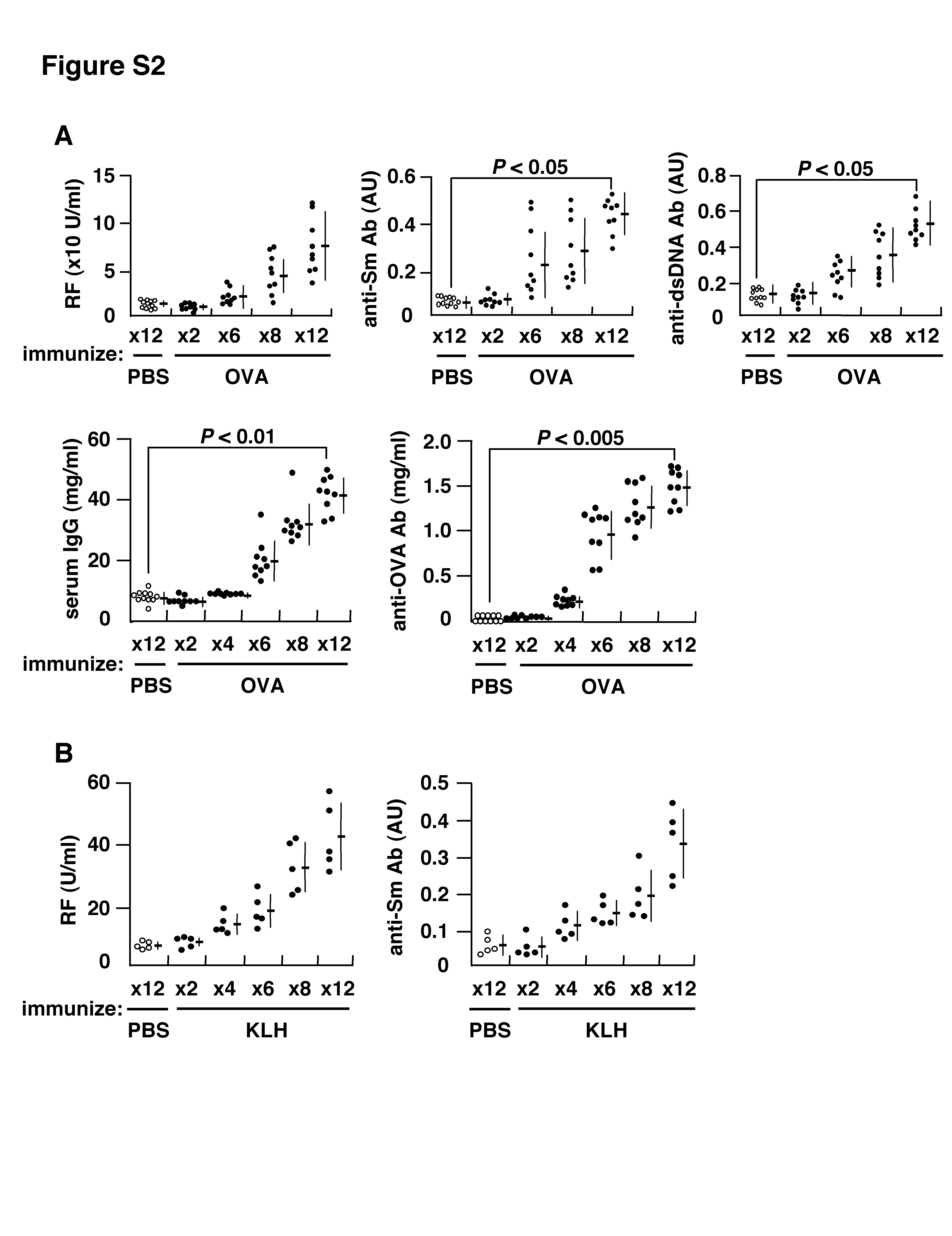

Supplement: Figure S2 — Generation of autoantibodies after repeated immunization with antigen. (A) The 8 week-old BALB/c mice were injected i.p. with 500 µg OVA every 5 d, and serum RF and anti-Sm, and anti-dsDNA antibodies (upper), and serum IgG and anti-OVA antibodies (lower) were quantified by ELISA 2 d after respective immunization. An arbitrary unit (AU) of 1.0 is the equivalent titer in sera of MRL/lpr mice. Serum IgG was quantified by ELISA (Bethyl Laboratories), and anti-OVA antibody was quantified using mouse anti-OVA monoclonal antibody (OVA-14; Sigma) as reference. (B) BALB/c mice were immunized i.p. with 100 µg KLH every 5 d. Serum RF and anti-Sm antibodies were measured by ELISA 2 d after respective immunization, AU 1.0 = equivalent detected in sera of MRL/lpr mice. (1.00 MB TIF) [file pone.0008382.s002.tif]

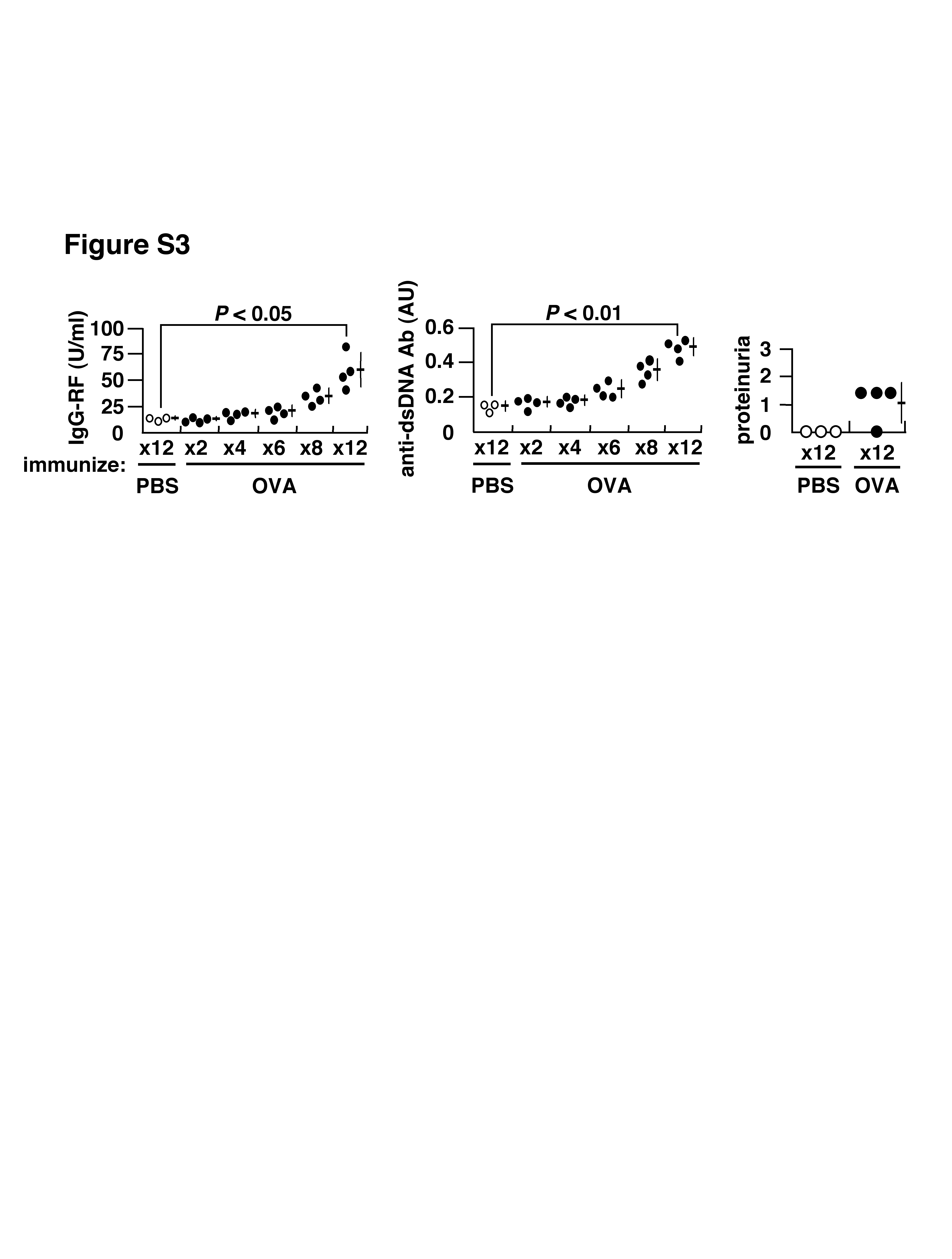

Supplement: Figure S3 — Induction of autoantibodies in CD8+ T cell-deficient mice. β2m-deficient mice were immunized with 500 µg OVA via i.p. injection every 5 d, and IgG-RF, anti-dsDNA antibody, and proteinuria were measured. (0.69 MB TIF) [file pone.0008382.s003.tif]

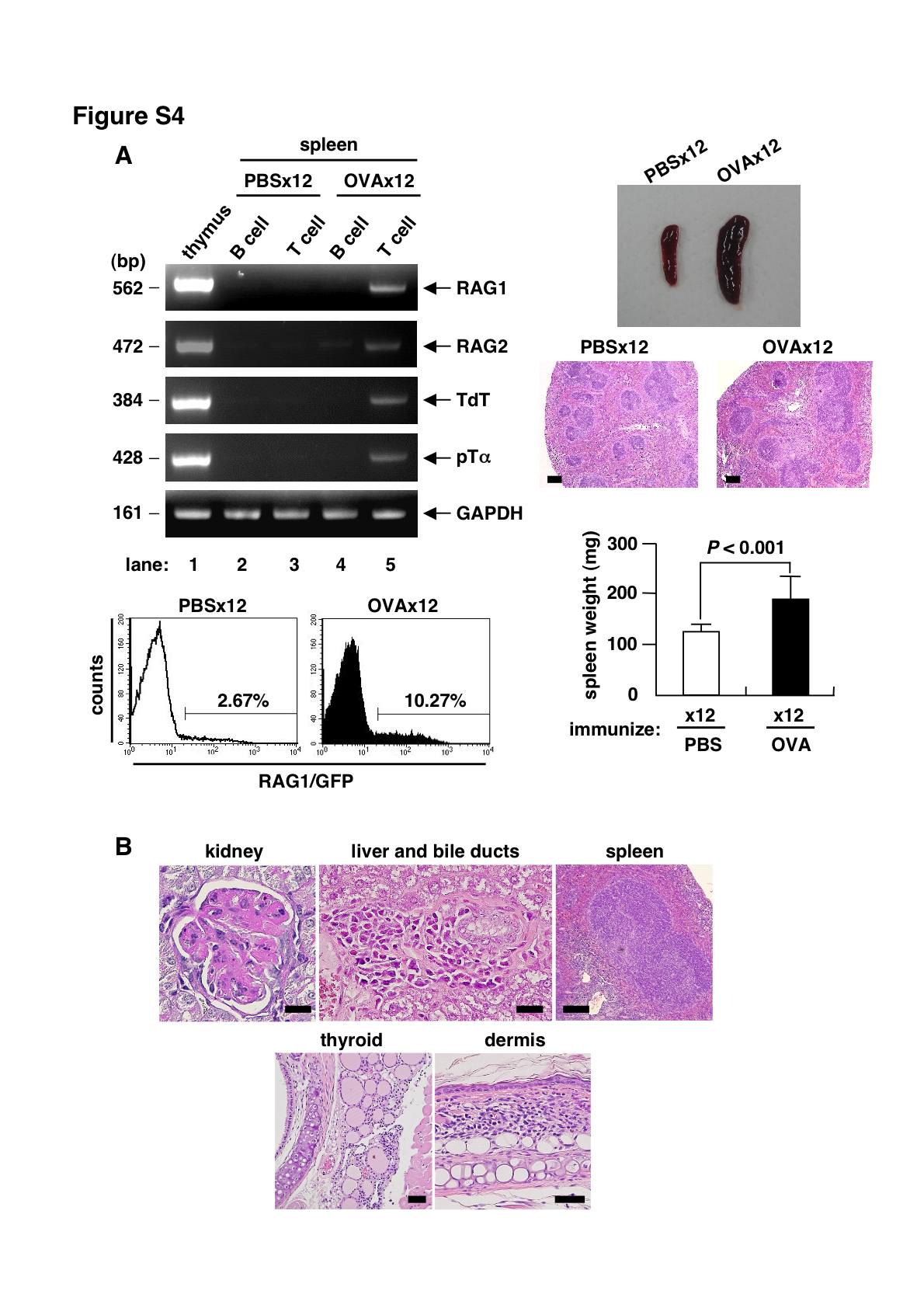

Supplement: Figure S4 — Expression of V(D)J recombinase complex and histopathology of OVA-immunized BALB/c mice. (A) Expression of V(D)J recombinase complex after immunization 12× with OVA as detected using RT-PCR (upper left). GFP+ cells in the CD4+ T cell of rag1/gfp knock-in mice after immunization 12× with OVA (lower left). Appearance and weights of spleens and a representative low-magnification view of the spleens from PBS- and OVA-immunized mice (right, mean ± SD, 9 mice/group). Enlarged lymphoid follicles with marked germinal centers were seen in mice immunized with OVA (H&E staining, bar = 200 µm; original magnification ×20). (B) Representative renal and extra-renal histopathology in the mice immunized 12× with OVA. A wire-loop-like massive membranous glomerulonephritis in the kidney (upper left) (PAS staining, bar = 20 µm; original magnification ×400), plasma cell infiltrates around bile ducts (upper middle) (bar = 20 µm; original magnification ×400), expansion of lymphoid follicle in the white pulp of spleen (upper right) (bar = 200 µm; original magnification ×40), focal infiltrates of mononuclear cells to thyroid (lower left) (bar = 50 µm; original magnification ×100), and diffuse infiltration of inflammatory cells into auricular subcutaneous tissue (upper right) (bar = 50 µm; original magnification ×200). (6.01 MB TIF) [file pone.0008382.s004.tif]

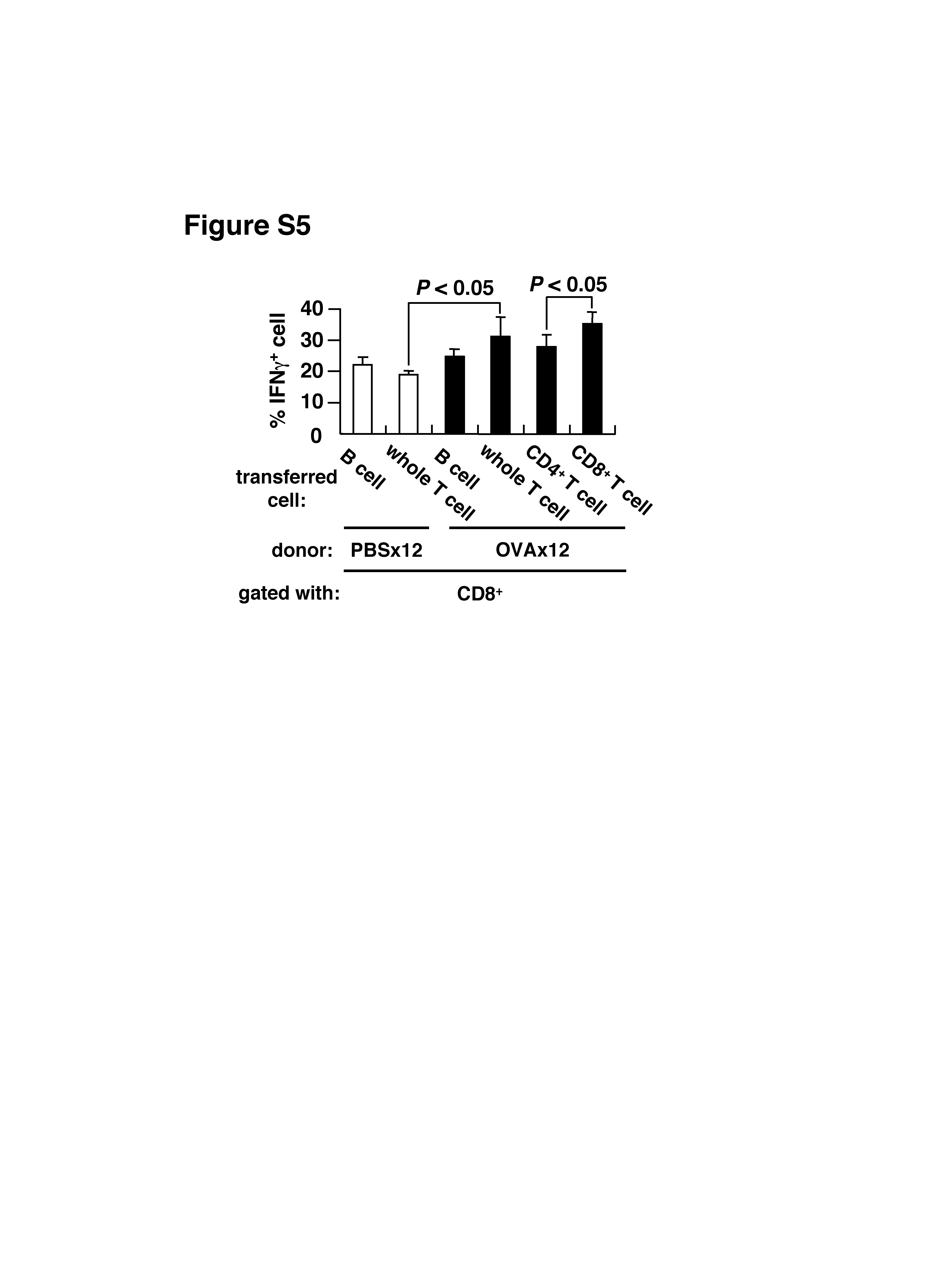

Supplement: Figure S5 — The de novo generation of IFNγ-producing CD8+ T cells in recipient mice after cell transfer. Percentage of IFNγ+ cells within the CD8+ T population of the recipient mice was examined 2 weeks after cell transfer (mean ± SD, 5 mice/group). (0.73 MB TIF) [file pone.0008382.s005.tif]

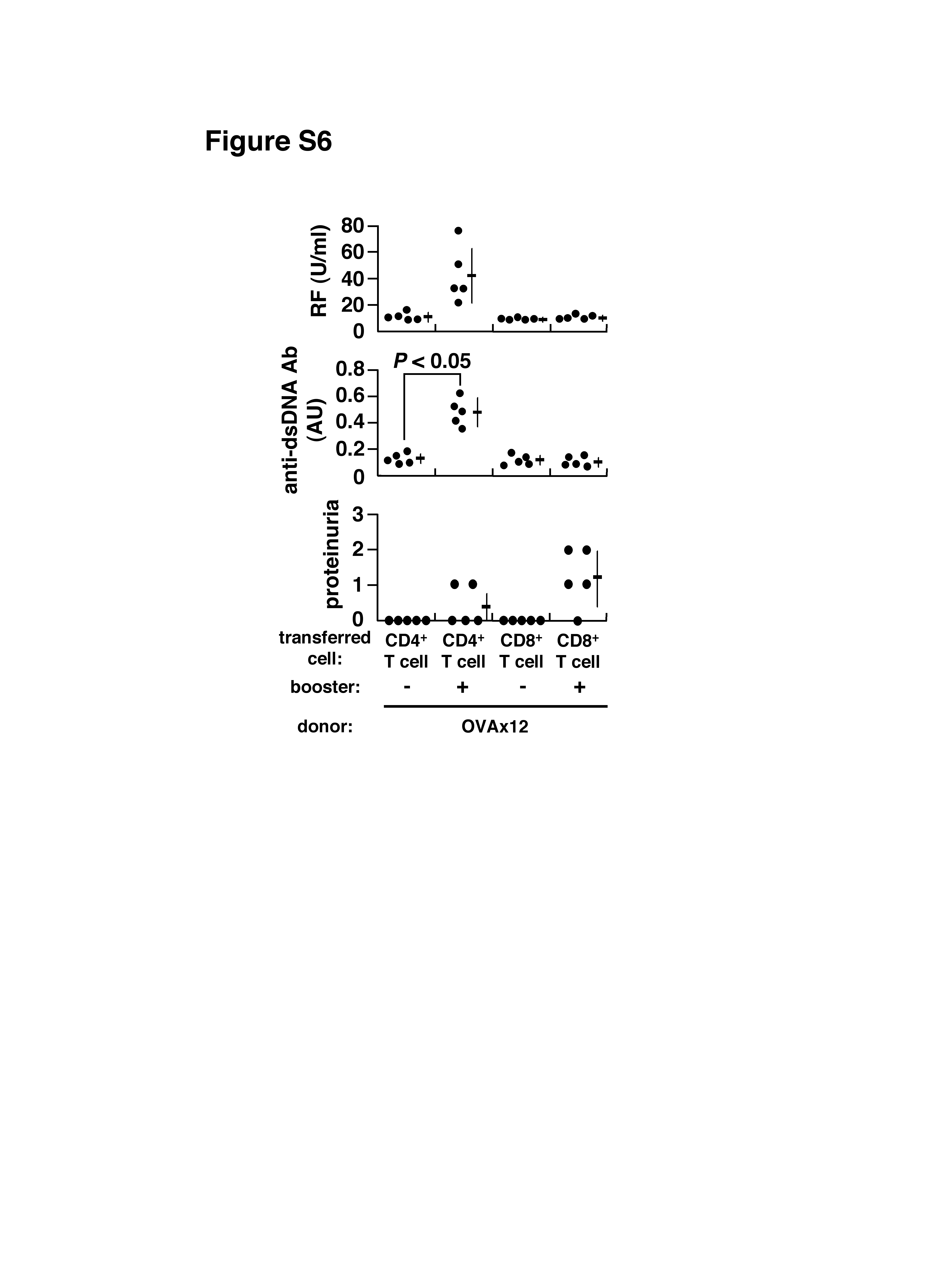

Supplement: Figure S6 — Transfer of the ability to induce anti-ds DNA antibody or tissue injury by transfer of CD4+ or CD8+ T cells, respectively. Adoptive transfer of cells from OVA-immunized mice into naïve BALB/c mice, with or without 1× booster injection of OVA (500 µg, 24 h post-transfer). Autoantibodies and proteinuria measured 2 weeks later. (0.70 MB TIF) [file pone.0008382.s006.tif]

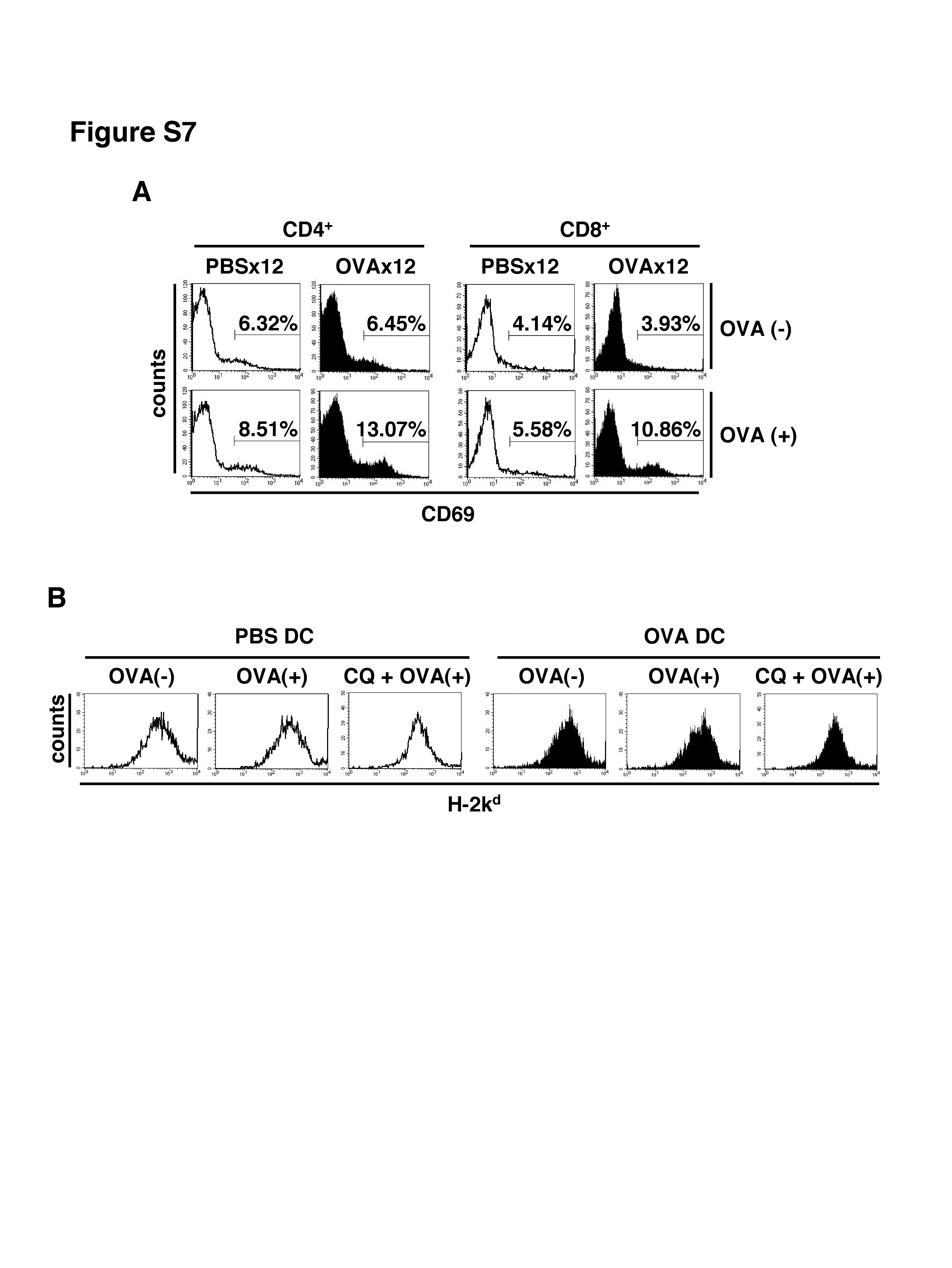

Supplement: Figure S7 — Antigen-specific activation of T cells and the expression of MHC class I on DC. (A) Spleen cells were cultured with or without 1 mg/ml of OVA for 24 h, and the expression of CD69 on CD4+ T or CD8+ T cells was examined by flow cytometry. (B) DC from PBS- or OVA-immunized mice (PBS DC or OVA DC) were incubated in the presence or absence of chloroquine (CQ) (20 µg/ml) for 2 h and OVA (1 mg/ml) for 3h. OVA- and/or CQ-pulsed DCs were stained with biotin-conjugated anti-H-2kd antibody (SF1-1.1; BD PharMingen) and PE-conjugated streptavidin (BioLegend). (1.62 MB TIF) [file pone.0008382.s007.tif]

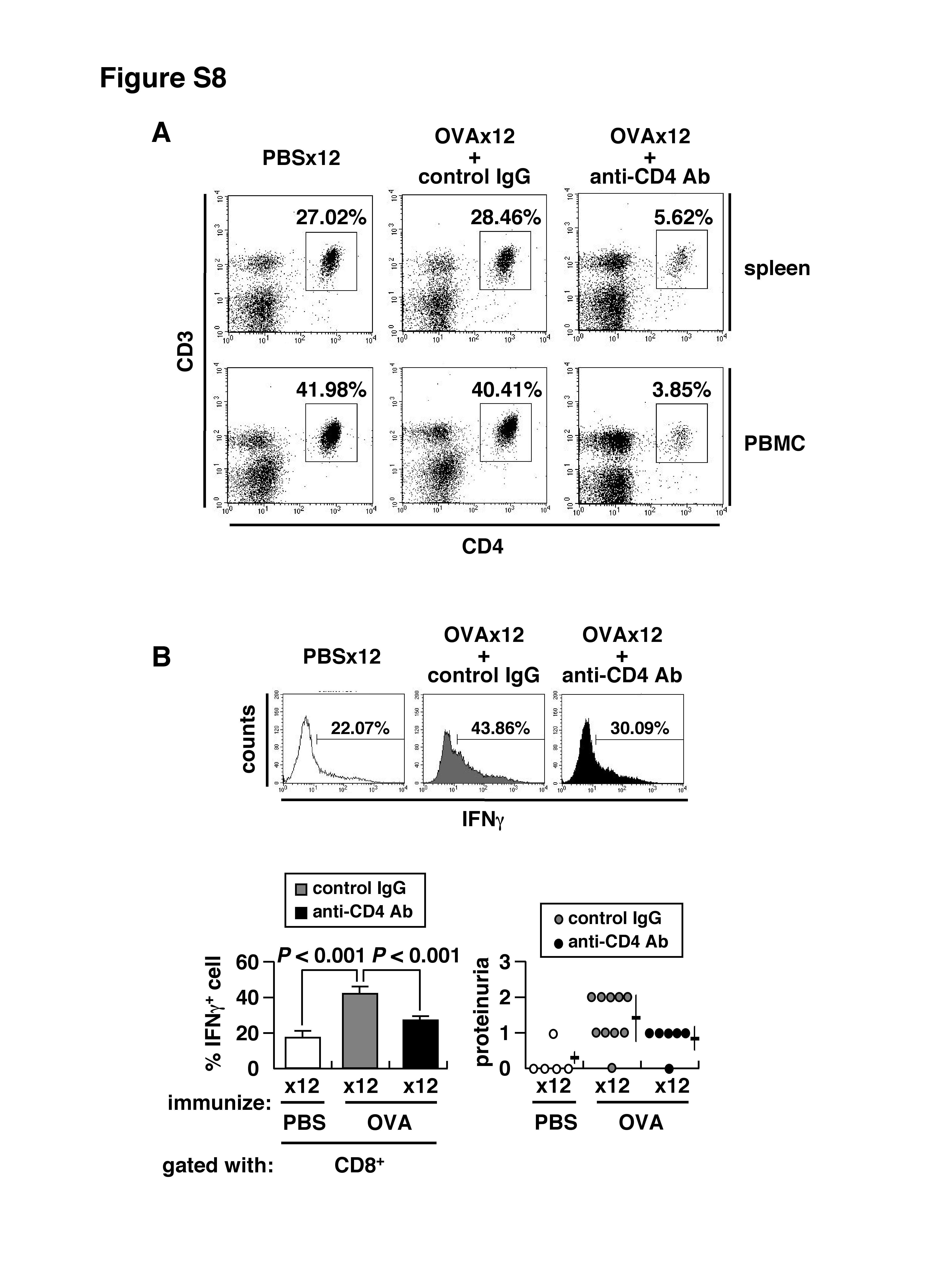

Supplement: Figure S8 — Requirement of CD4+ T cell help for inducing autoimmune tissue injury. The mice were depleted of CD4+ T cells by treatment with 200 µg anti-CD4 antibody (Ab) (GK1.5; BioLegend) 24 h prior to 6×, 9× and 12× immunization with OVA. Control mice were injected with 200 µg rat IgG (CALTAG Lab.). (A) A representative flow cytometry plot showing that CD4+ T cells were depleted to 5.56±2.30% in the spleen and 3.42±1.02% in peripheral blood mononuclear cells (PBMC) 9 d after 3rd treatment with anti-CD4 Ab. (B) Mice were immunized 12× with OVA with or without adding anti-CD4 antibodies, and the number of IFNγ+ cells within the CD8+ T population (upper and lower left) (mean ± SD, 5 mice/group) and proteinuria (lower right) were evaluated. (2.02 MB TIF) [file pone.0008382.s008.tif]

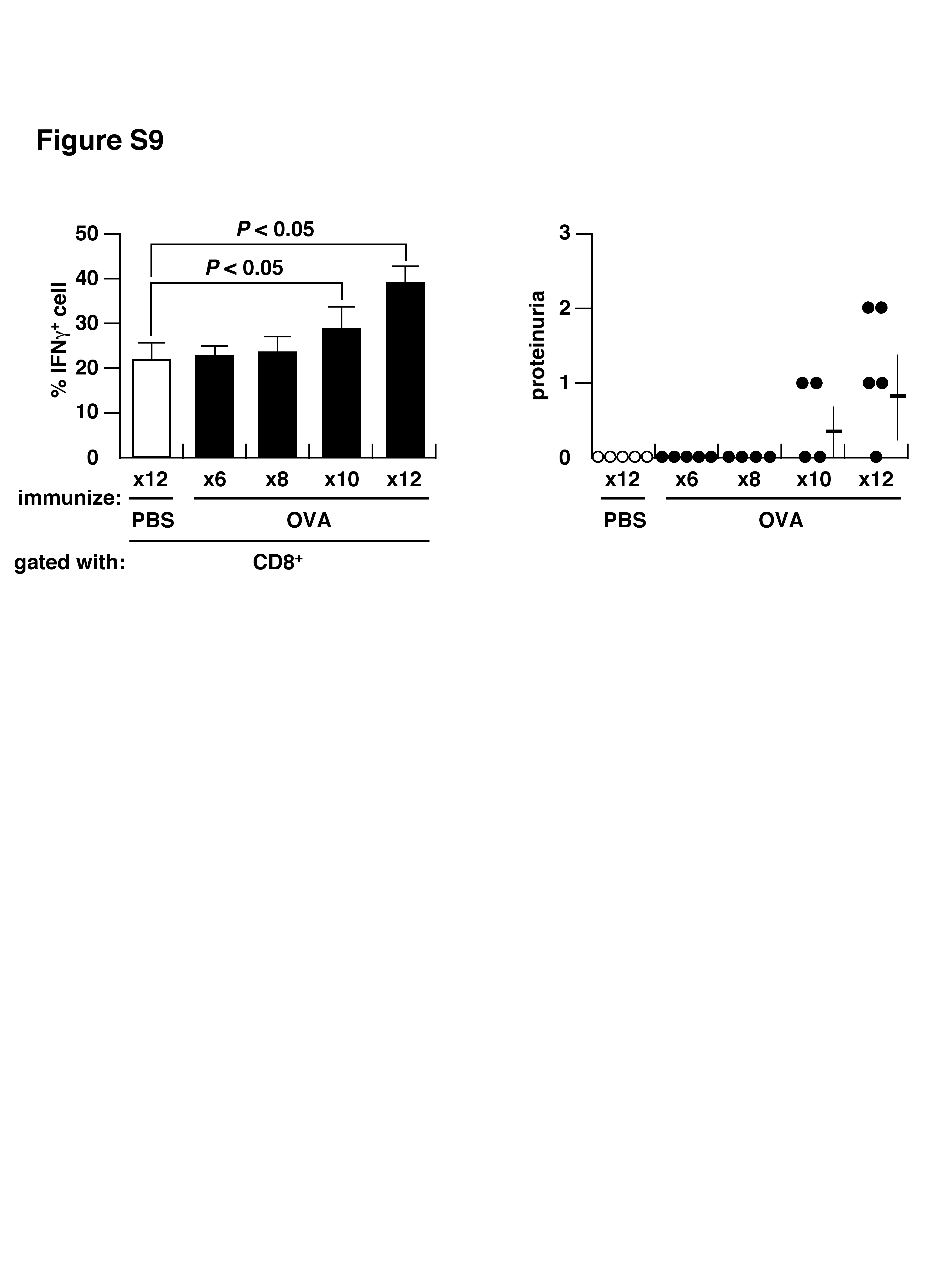

Supplement: Figure S9 — Study on the requirement of autoantibody-inducing CD4+ T cells for autoimmune tissue injury. Neither OVA-specific matured IFNγ+CD8+ T cells or autoimmune tissue injury were observed until BALB/c mice were immunized at least 10× with OVA. The percent splenic IFNγ+CD8+ T cells (left, mean ± SD, 4 or 5 mice/group) and proteinuria (right) were examined after immunization 6×, 8×, 10× and 12× with OVA. (0.67 MB TIF) [file pone.0008382.s009.tif]
